# Epigenetic landscape reveals *MEF2* and *SIX* family-mediated transcriptional networks underlying dietary NFC/NDF ratio-induced muscle development and meat quality in Tibetan sheep

**DOI:** 10.3389/fnut.2025.1658319

**Published:** 2025-10-15

**Authors:** Rengeerli Sa, Fengshuo Zhang, Zhenglu Yang, Chengdi Shi, Shengzhen Hou, Song Zhang, Linsheng Gui

**Affiliations:** ^1^College of Agriculture and Animal Husbandry, Qinghai University, Xining, China; ^2^Shenzhen Branch Guangdong Laboratory for Lingnan Modern Agriculture, Key Laboratory of Livestock and Poultry Multi-Omics of MARA, Agricultural Genomics Institute at Shenzhen, Chinese Academy of Agricultural Sciences, Shenzhen, China

**Keywords:** NFC/NDF ratio, ATAC-seq, H3K27ac CUT&Tag, transcriptomics, Tibetan sheep, muscle

## Abstract

**Introduction:**

We investigated epigenetic mechanisms underlying muscle development in black Tibetan sheep fed different dietary ratios of non-fiber carbohydrate to neutral detergent fiber (NFC/NDF), integrating phenotypic analyses (growth performance, meat quality, muscle histomorphology) with multi-omics approaches (ATAC-seq, H3K27ac CUT&Tag, RNA-seq).

**Methods:**

The subjects were 2-month-old weaned male Tibetan sheep with an initial body weight of 10.45 ± 0.96 kg. Using a completely randomized study design, 90 sheep were randomly assigned to three treatment groups with 30 sheep per group. Each treatment group consisted of 5 pens with 6 sheep per pen. The dietary NFC/NDF ratios of these three treatment groups were 0.71 (Group L), 1.16 (Group M) and 1.82 (Group H).

**Results:**

An NFC/NDF ratio of 1.82 yielded optimal growth performance with increased live weight, carcass weight, and dressing percentage. This ratio significantly improved meat tenderness, water-holding capacity, and pH values while reducing hardness and chewiness, accompanied by compact muscle fiber architecture. Epigenomic analyses revealed epigenetic signals predominantly localized within distal intergenic regions (10–100 kb). Enhancers showed dynamic responsiveness to dietary NFC/NDF changes, with *ME*F2 and *SIX* transcription factor binding sites enriched in upregulated enhancers. RNA-seq confirmed functional associations between epigenetic modifications and gene expression, revealing enrichment of fatty acid metabolism, epithelial cell proliferation, and macrophage chemotaxis pathways. Differential expression of key genes (*EGR3, FGF7, HPGD, C5, EDNRB*) corroborated *MEF2* and *SIX* families’ central role in nutrient-mediated muscle remodeling.

**Discussion:**

These findings elucidate how dietary NFC/NDF ratios optimize muscle development through enhancer activation, providing insights for precision nutrition strategies in livestock production.

## Graphical abstract

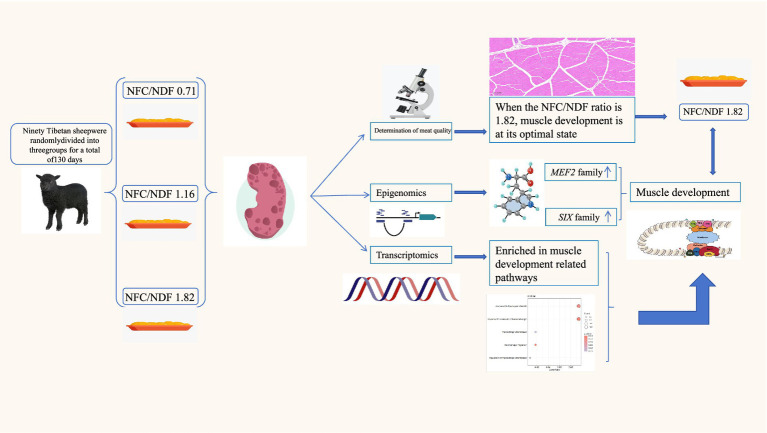

## 1 Introduction

Meat is a crucial source of protein for humans, directly impacting their health and quality of life (1). Among the different available types of meat, lamb is especially popular with Chinese consumers due to its high protein content and low fat and cholesterol content. As concerns about nutrition and safety grow, the demand for high-quality lamb has increased steadily (2). The proportion of non-fibrous carbohydrates (NFC) and neutral detergent fiber (NDF) in animal diets is known to significantly influence their muscle growth, lean meat yield, and meat flavor and fiber structure (3). Tibetan sheep are known for their resistance to stress and superior meat quality. Their unique growth environment makes them an ideal model for investigating the molecular mechanisms underlying the effects of varying dietary NFC/NDF ratios on muscle phenotypic traits (4). According to genome-wide association studies, most single-nucleotide polymorphisms (SNPs) related to muscle quality traits are found within non-coding regions in Tibetan sheep, particularly in the regulatory elements (5). For example, enhancers have been found to modulate gene expression in different tissues at various time points by altering chromatin configuration and specific histone modifications, thereby influencing development and other complex biological processes (6). A comprehensive annotation of these enhancers could help us understand the potential regulatory mechanisms through which dietary NFC and NDF affect muscle phenotypes and meat quality in livestock.

Enhancers are non-coding DNA sequences located both upstream and downstream of transcription start sites (TSS). They regulate the transcriptional activity of genes by facilitating their interactions with transcription factors (7). One key feature of enhancers is their ability to influence gene expression regardless of their position and direction. These elements are typically located within regions of open chromatin and are essential for transcription factor binding and gene regulation (8). Enhancers are enriched with histone modifications, such as H3K27ac, demonstrating their role as active regulatory elements (9). RNA sequencing (RNA-seq) can provide key insights into the target genes regulated by enhancers, allowing the identification of gene expression alterations under specific conditions. This can reveal which genes are potentially modulated by specific enhancers (10). Meanwhile, the Assay for Transposase-Accessible Chromatin with high-throughput sequencing (ATAC-seq) is an effective method for detecting open chromatin regions (11). Additionally, CUT&Tag, which targets histone modifications of active enhancers (e.g., H3K27ac), enables the precise identification of these regions (10). The combination of these three techniques facilitates the systematic identification of enhancers and provides information about their roles in gene regulatory networks and their influence on gene transcription. In livestock research, the enrichment of active enhancer signals has been used to predict gene expression in sheep macrophages and uncover phenotype-related mutations (12). This suggests that enhancers play a critical role in regulating core genes involved in livestock production.

However, current research on how dietary nutrients influence muscle phenotype and meat quality through epigenetic mechanisms in livestock is still limited. Most studies have focused on analyzing the direct effects of dietary components on muscle growth and meat quality, while the underlying molecular regulatory mechanisms, especially the enhancer-mediated transcriptional regulatory networks, remain largely unknown. Given the critical role of histone modifications in gene expression regulation, an in-depth investigation of the changes in histone modifications of muscle tissue enhancers induced by feeding different NFC/NDF ratios is expected to reveal the key regulatory mechanisms of muscle traits and meat quality formation in Tibetan sheep.

This investigation aims to elucidate the epigenomic heterogeneity in Tibetan sheep subjected to varying dietary ratios of non-fibrous carbohydrates (NFC) and neutral detergent fiber (NDF) through the integration of multi-dimensional data, encompassing phenotypic parameters (growth performance, meat physicochemical characteristics, and muscle histomorphology) and high-throughput molecular characterization techniques (RNA-seq, ATAC-seq, and CUT&Tag). Particular emphasis is placed on elucidating the functional role of epigenetic markers, notably H3K27ac, in the regulation of myogenesis and metabolic processes. Through the comprehensive application of multi-omics approaches, this research endeavors to delineate the regulatory mechanisms by which differing NFC: NDF dietary ratios modulate muscle quality in Tibetan sheep, thereby establishing a theoretical foundation for enhancing feeding efficiency and meat quality, ultimately contributing to the advancement of precision livestock production.

## 2 Materials and methods

### 2.1 Animal ethics

The study was conducted in accordance with the ARRIVE guidelines (AVMA Guidelines for the Euthanasia of Animals: 2020 Edition) and approved by the Animal Care Committee of Qinghai University (approval number: QUA-2020-0710, approved on July 10, 2020). All experimental procedures involving animals were performed in compliance with institutional guidelines and the laboratory animal management and welfare regulations established by the Ethics Committee of Qinghai University, Xining, Qinghai, China. The study protocols were designed to minimize animal suffering and reduce the number of animals used.

### 2.2 Experimental design and sample collection

The experiment was carried out from March 1, 2021, to July 6, 2021, at the Heihuang Sheep Breeding Center in Guinan County, Qinghai Province, China. The subjects were 2-month-old weaned male Tibetan sheep with an initial body weight of 10.45 ± 0.96 kg. Using a completely randomized study design, 90 sheep were randomly assigned to three treatment groups with 30 sheep per group. Each treatment group consisted of 5 pens with 6 sheep per pen. The dietary NFC/NDF ratios of these three treatment groups were 0.71 (Group L), 1.16 (Group M) and 1.82 (Group H). The sheep diet consisted of roughage (oat silage and oat hay in a 1:1 dry matter ratio). The composition of the diets for each treatment group is shown in Table 1. Feed was provided twice daily at 08:30 and 16:30, and the sheep were allowed ad libitum access to feed and water. At the end of the experimental period, three sheep from each group were randomly selected from different pens to ensure representative sampling across pens. The selected sheep were transported to the local abattoir, held in lairage for 12–24 h with access to water but no feed, and subsequently slaughtered according to standard commercial procedures. Immediately after slaughter (within 30 min post-mortem), the Longissimus lumborum between the 12th and 13th ribs was collected from all slaughtered sheep. Each muscle sample was divided into three portions: the first portion was immediately used for pH and texture analysis on fresh muscle; the second portion was fixed in formalin for histological analysis; and the third portion was snap-frozen in liquid nitrogen and stored at −80 °C for subsequent molecular analysis.

**Table 1:** Ingredient and chemical composition of experimental diets on DM basis.

Items	L	M	H
Ingredients (%)			
Oaten hay	35	25	15
Oat silage	35	25	15
Corn	16.14	30.06	40.50
Rapeseed meal	2.28	7.45	14.13
Cottonseed meal	2.22	3.10	6.39
Soybean meal	2.16	2.19	2.23
NaCl	0.80	0.80	0.80
Limestone	0.80	0.80	0.80
NaHCO3	0.10	0.10	0.10
CaHPO4	0.50	0.50	0.50
Premix1	5.00	5.00	5.00
Analyzed composition			
DE (MJ/kg)	12.58	12.61	12.65
Crude protein %	13.78	13.82	13.86
Ash %	4.65	4.64	4.62
Ether extract %	3.91	4.95	4.01
Starch %	16.28	23.46	28.35
Neutral detergent fiber %	45.14	36.26	26.33
Acid detergent fiber %	29.17	22.68	16.15
Non-fiber carbohydrates%	32.05	42.06	47.92
Non-fiber carbohydrates/Neutral detergent fiber	0.71	1.16	1.82
Ca %	1.13	1.12	1.12
P %	0.24	0.24	0.23

Provided per kilogram of the diets: Cu 15 mg, Fe 55 mg, Zn 25 mg, Mn40 mg, Se 0.30 mg, I 0.5 mg, Co 0.20 mg, VA 20,000 IU, VD 4,000 IU, and VE 40 IU. Digestive energy is the calculated value, while the rest are measured values.

### 2.3 Assessment of growth performance

The live weight of each sheep was determined at the conclusion of the experimental period. Following this, a standardized slaughter protocol was implemented, whereby the head, hooves, and visceral organs were removed to obtain the bone-in carcass. Carcass weights were precisely quantified, and the dressing percentage (DP) was calculated according to the following equation: DP (%) = (carcass weight/live weight) × 100.

### 2.4 Experimental design and sample collection

Longissimus lumborum muscle samples were excised from the region between the 12th and 13th ribs, and pH measurements were conducted using a calibrated portable pH meter (PHBJ-260, Shanghai, China). The pH electrode was inserted to a depth of 2 cm into the muscle tissue. Initial pH values were recorded within 45 min post-mortem, and ultimate pH values were determined at 24 h post-mortem. Prior to measurements, the pH meter was calibrated using standard buffer solutions (pH 4.01 and 6.86) in a two-point calibration procedure to ensure analytical precision. Texture profile analysis was performed using a CT3 texture analyzer (Brookfield, USA) to assess the hardness and chewiness of cubic samples (1 cm^3^× 1 cm^3^× 1 cm^3^) oriented parallel to the muscle fiber direction. To determine thawing loss, meat samples were thawed at 4 °C for 12 h, after which surface exudate was gently blotted with filter paper prior to accurate weighing. Thawing loss percentage was calculated using the equation: Thawing loss (%) = ((pre-freezing weight - post-thawing weight) / pre-freezing weight) × 100. Thawed meat samples were subjected to thermal processing in an 85 °C water bath for 20 min, subsequently cooled to ambient temperature, surface moisture was removed with absorbent paper, and samples were weighed. All cooking was performed in a single batch to eliminate batch effects. Samples were randomly allocated within the cooking vessel to ensure uniform heat exposure. Cooking loss percentage was determined using the formula: Cooking loss (%) = ((pre-cooking weight - post-cooking weight) / pre-cooking weight) × 100.

### 2.5 Experimental design and sample collection

The histological analysis of the Longissimus lumborum muscle was performed at Cisco Science Biotechnology Co., Ltd. (Tianjin, China). Hematoxylin and eosin (HE) staining was conducted in accordance with the protocol described by Zhang et al. (13). Initially, the sections were dewaxed and processed using a series of laboratory equipment, including a dehydration machine and an embedding station. During the staining process, the sections were first treated with hematoxylin, which stained the nuclei blue. After dehydration through a gradient of alcohol concentrations, the sections were stained with eosin, which turned the cytoplasm red. After staining, the sections were cleared, mounted in neutral resin for long-term preservation and microscopic analysis, and examined using a Nikon Eclipse E100 upright optical microscope (Nikon, Japan).

### 2.6 Experimental design and sample collection

#### 2.6.1 Sequencing library preparation

Nuclei were extracted from frozen muscle cells using a previously reported method, and ATAC-Seq (N256A, Novoprotein) and CUT&Tag (N259-YH01-01A, Novoprotein) libraries were constructed using commercial kits. Each CUT&Tag assay system required 3 μg of the anti-H3K27ac antibody (ab4729, Abcam). High-throughput sequencing was performed using the Nova-PE150 strategy.

#### 2.6.2 Data processing

Adapter sequences and low-quality reads were eliminated using Trim Galore software. Read mapping was performed using Bowtie2 software. Low-quality aligned and duplicate reads were removed using Sambamba software. Subsequently, bam files were converted into bigWig files using the bedtools bamCoverage tool to visualize the enrichment of the read signal. Finally, macs2 software was employed to call peaks. The 100-bp interval before and after each peak summit was used for the enrichment analysis of transcription factor motifs using HOMER software.

### 2.7 Identification and merging of idr peaks

Within-group peaks were processed using idr software to obtain consistent within-group peaks, which were referred to as idr peaks. The acquisition of merged peaks was divided into three steps: first, samtools software was employed to merge the bam files of all samples; then, macs2 software was employed to call peaks for the merged bam file. Finally, the bedtools intersect tool was employed to select the peaks that overlapped with idr peaks, with the parameter set to -F 0.5.

Peak signal calculation and identification of cis-regulatory elements (CREs).

A read count matrix of peaks was generated using featureCounts software. The read count matrix was normalized (TPM) and corrected for library size using edgeR software to obtain the final peak signal matrix. Based on the annotation files of the Tibetan sheep genome in the Ensembl database, the location files of the regions adjacent (1 kb upstream and 500 bp downstream) to TSSs were generated. Dynamic peaks were defined as the top 5,000 enhancers showing highest variance (coefficient of variation >0.5) across groups L, M, and H. Promoters were classified as H3K27ac/ATAC-seq overlapping peaks within TSS ± 1.5 kb; enhancers were peaks beyond this range.

### 2.8 Functional enrichment analysis

Based on the Genomic Regions Enrichment of Annotations Tool (GREAT) principle, the regulatory regions of genes (5 kb upstream and 1 kb downstream of TSSs) were identified. Genomic regions overlapping with the regulatory regions were then assigned to the corresponding genes. If the regulatory regions showed no overlapping genomic regions, the closest genomic regions (<1 Mb) were assigned to the corresponding genes. The functional annotation of sheep genes was performed using eggnog-mapper software. Subsequently, functional enrichment analysis was performed on the associated genes using the AnnotationForge and clusterProfiler R packages.

### 2.9 Transcriptomic analysis

Total RNA was extracted from the Longissimus lumborum muscle of Tibetan sheep (n = 3) using TRIzol reagent (Thermo Scientific, MA, USA). The concentration and quality of RNA were evaluated using the Agilent 2,100 (Agilent, CA, USA) and NanoDrop 2000 (Thermo Scientific, MA, USA) systems to confirm the suitability of RNA samples for downstream library construction. Ribosomal RNA (rRNA) was selectively removed to retain all coding and non-coding RNAs, as recommended by prior RNA-seq workflows [e.g., (14)]. The remaining RNA was randomly fragmented and used as a template to synthesize first-strand cDNA with random hexamer primers. Buffer, dNTPs, RNase H, and DNA Polymerase I were subsequently introduced to facilitate the synthesis of the second cDNA strand. Enzymatic treatment with UNG (Uracil-N-Glycosylase) was performed, followed by end repair, A-tailing, and sequencing adaptor ligation. The resulting 200-bp fragments were isolated through agarose gel electrophoresis and amplified via PCR to construct the sequencing library.

The cDNA library was prepared using the NEB#7530 kit (New England Biolabs, CA, USA), quality-checked, and sequenced on the Illumina HiSeq 2,500 platform at Gene Denovo Biotechnology Co., Ltd. (Guangzhou, China). Sequencing data quality was assessed using fastp (v0.18.0), with low-quality bases and adaptors trimmed using the FASTX toolkit. High-quality sequences were mapped to the *Ovis aries* (Tibetan sheep) reference genome (ASM1117029v1) using TopHat2 (v2.1.1). Differential gene expression was identified in terms of FPKM, and a comparative analysis across three groups was conducted using Cuffdiff. The functional annotation of differentially expressed genes (DEGs) was performed using the Gene Ontology (GO) database. Enrichment analysis was conducted using the GOseq R package and KOBAS software by applying a significance threshold of FDR ≤ 0.05. To validate the reliability of the RNA-Seq data, five genes were selected for quantitative real-time PCR (qRT-PCR) analysis, with glyceraldehyde-3-phosphate dehydrogenase (*GAPDH*) serving as the internal reference gene. All primer sequences utilized in this study are presented in Table 2.

**Table 2:** Primers used in RT-qPCR.

Name	Gene ID	Primer sequence (5′-3′)	Product length (bp)
EDNRB	443,139	L-ATGATGGAGACCCCGACTGA	103
		R-CCACCATCCTTCCCCCTCTA	
C5	101,121,825	L-AGGGGATCTGTTCATCGGGA	115
		R-AGCACGGTGAAGGTAACCAG	
HPGD	101,112,958	L-TGGTGTGAGACTGAACGCAA	157
		R-GGCAATCATCGAAGGGTCCA	
FGF7	443,095	L-CCCCGAGCGACATACAAGAA	174
		R-CCAACAGCCACTGTCCTGAT	
EGR3	101,121,578	L-ACAGCAACCTCTTCCCCATG	141
		R-TGGTCTCCAGCGGGGTAATA	
GAPDH	443,005	L-ACCTGCCGCCTGGAGAAAC	120
		R-TGGTCCTCAGTGTAGCCTAGAATG	

The table lists the primer design information of quantitative real-time PCR for six genes, including gene name, gene ID, sequence of forward primer (L) and reverse primer (R) (5′-3′) and expected amplification product length. *EDNRB*, *C5, HPGD, FGF7,* and *EGR3* were the target genes, and *GAPDH* was the internal reference gene. The amplified products of all primers were in the range of 103–174 BP, which was suitable for QRT PCR detection.

### 2.10 Data analysis

Statistical analyses were performed using R software (version 4.2.1). Differences among the three dietary groups (L, M, and H) were assessed using one-way ANOVA followed by Tukey’s *post hoc* test with significance set at *p* < 0.05. For ATAC-Seq and H3K27ac CUT&Tag data, peak calling was performed using macs2, and the irreproducible discovery rate (IDR) method was applied to identify consistent peaks within groups. Peak signals were normalized to transcripts per million (TPM) and corrected for library size using edgeR. The STEM software was used to identify significantly enriched peak profiles across the three groups. Transcription factor motif enrichment analysis was conducted using HOMER with a 100-bp interval around peak summits. For RNA-Seq data, clean reads were mapped to the Tibetan sheep reference genome using TopHat2, and transcript abundance was quantified as FPKM using Cufflinks. Differentially expressed genes were identified using Cuffdiff (fold change ≥ 2, *p* < 0.05) and functionally annotated using the Gene Ontology database. The integration of epigenomic and transcriptomic data was performed by assigning peaks to their potential target genes using the GREAT approach. We performed comprehensive multi-omics association analysis to explore correlations both within and between different omics layers. The analytical framework consisted of two complementary visualization approaches: Intra-omics correlations were visualized using heatmaps to display pairwise correlation coefficients among all phenotypic traits, revealing the interconnected nature of morphological and physiological characteristics. Inter-omics correlations were represented through network graphs, illustrating the associations between differentially expressed genes (DEGs) and phenotypic traits. This network-based approach enabled the identification of key gene-trait relationships and potential regulatory connections. Spearman’s rank correlation coefficient was employed to assess all gene-trait associations, accounting for potential non-linear relationships in the data. Statistical significance was determined using adjusted *p*-values to control for multiple testing. All correlation analyses and visualizations were performed using R software (version 4.3.0) with relevant packages including corrplot for heatmaps and igraph for network construction.

## 3 Results

### 3.1 Assessment of growth performance

**Figure 1:**
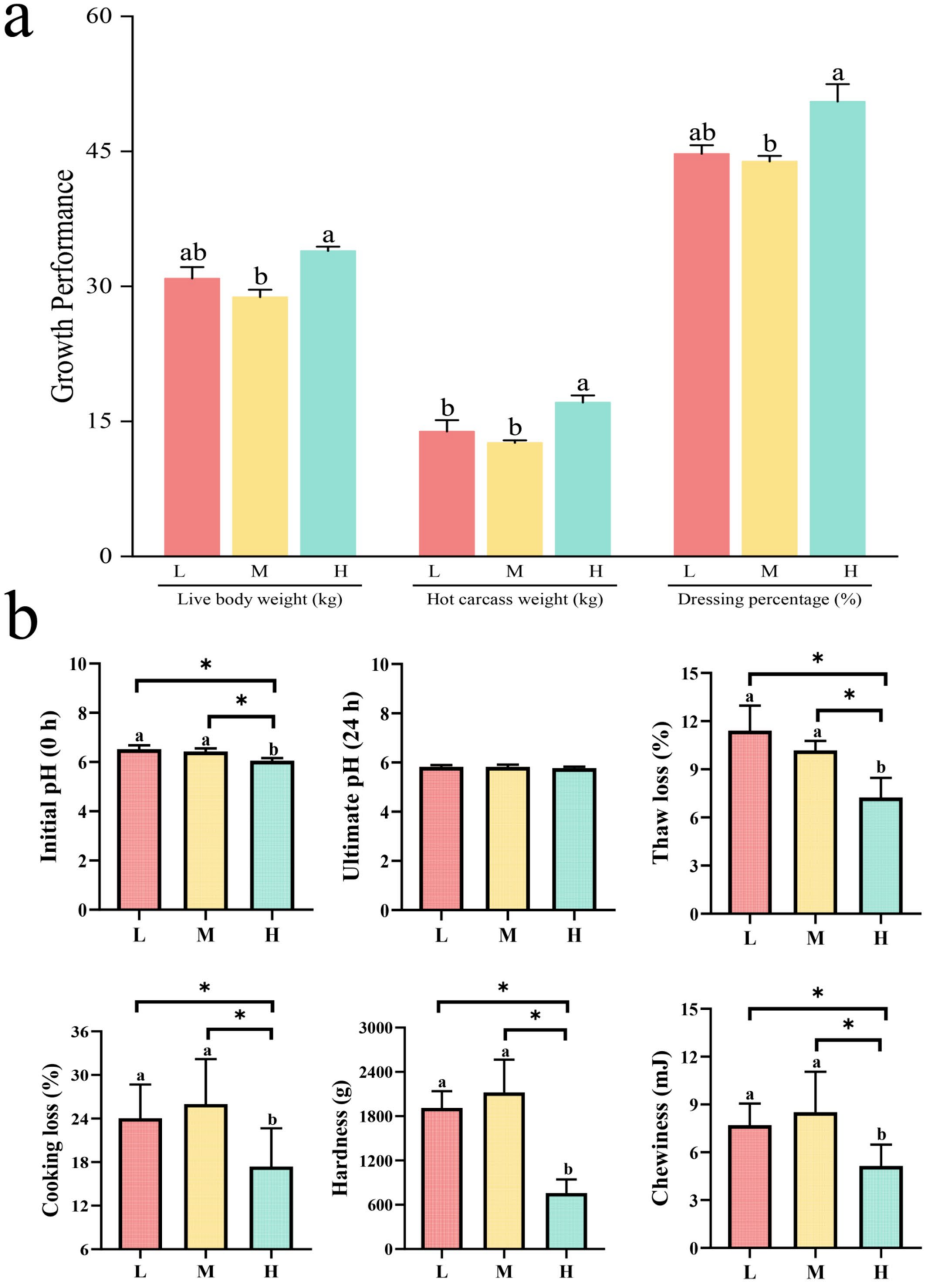
Bar graphs comparing growth performance and meat quality in three categories: L, M, and H. Graph **(a)** shows live body weight, hot carcass weight, and dressing percentage, with H generally higher. Graph **(b)** illustrates initial and ultimate pH, thaw loss, cooking loss, hardness, and chewiness, with significant differences among categories marked by asterisks.

Different proportions of non-fibrous carbohydrates (NFC) and neutral detergent fiber (NDF) exerted significant effects on the growth performance of Tibetan sheep (Figure 1a). Results indicated that sheep in group H exhibited significantly higher live weight and slaughter rate compared to group M (*p* < 0.05). Additionally, the carcass weight in group H was significantly greater than that observed in both groups M and L (*p* < 0.05).

### 3.2 Assessment of meat physicochemical properties

Assessment of meat physicochemical characteristics revealed (Figure 1b) that groups L and M exhibited significantly higher values for initial pH (0 h), cooking loss, thawing loss, chewiness, and hardness parameters compared to group H (*p* < 0.05).

### 3.3 Assessment of growth performance

**Figure 2:**
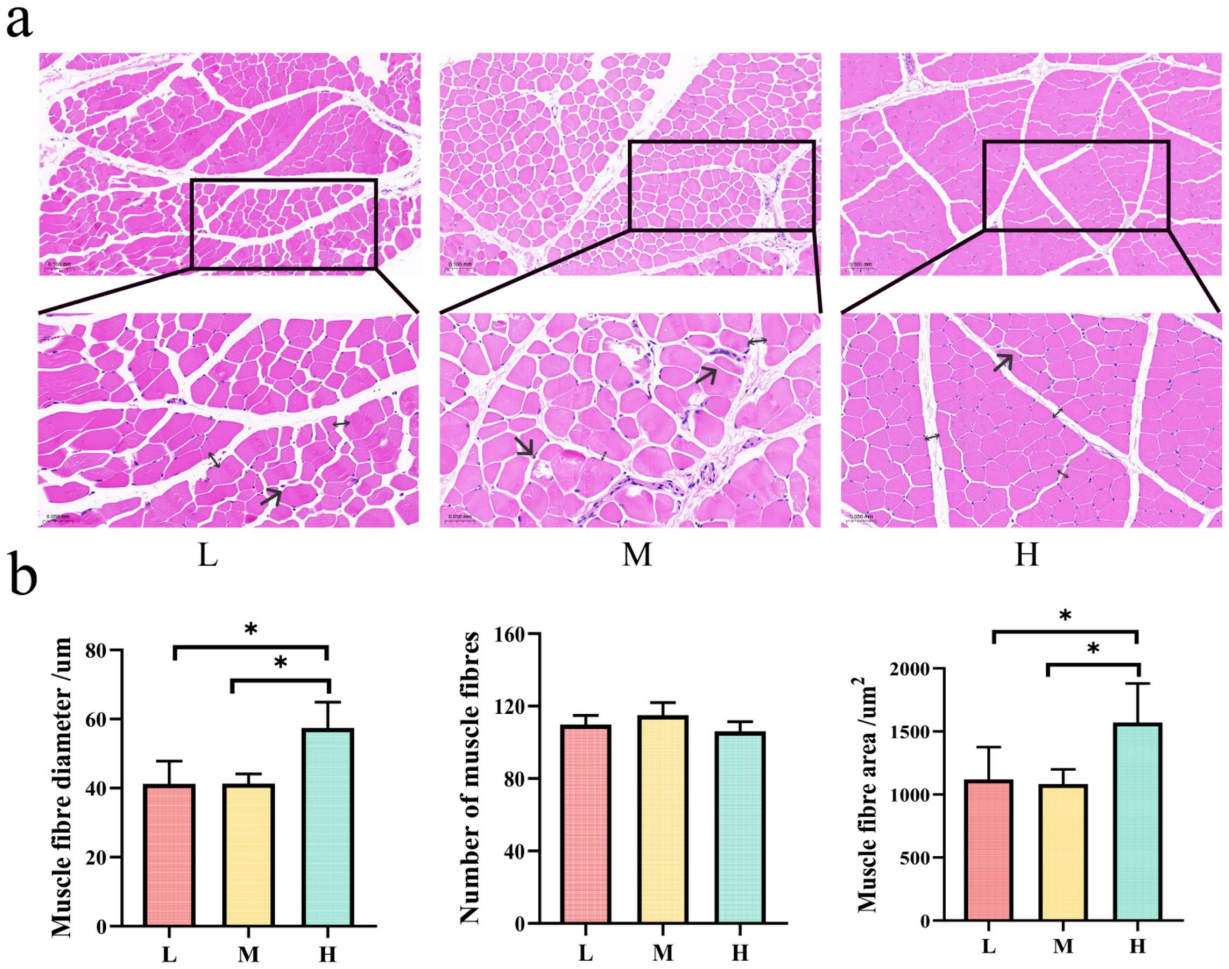
Panel **(a)** shows histological images of muscle fiber cross sections for conditions L, M, and H, with insets highlighting differences in morphology. Arrows (→) indicate fiber rupture; double arrows (↔) denote intermuscular spacing. Panel **(b)** presents bar graphs comparing muscle fiber diameter, number, and area across L, M, and H. Significant differences are indicated with asterisks, showing variations in muscle fiber diameter and area, with H having the largest values.

The histological examination of muscle cross-sections revealed distinct morphological patterns among the L, M, and H groups (Figures 2a,b). The H group displayed the most favorable muscle fiber characteristics, including an orderly fiber arrangement, clear fascicular structure, and well-defined perimuscular contours. Moreover, the muscle fibers in this group showed a uniform size distribution and adequate intermuscular spacing, indicating optimal muscle development. In contrast, the L group exhibited a relatively irregular fiber arrangement, variable fiber sizes, and less distinct fascicular structures, thus demonstrating poorer tissue organization than the H group. The M group exhibited fiber rupture and excessively large intermuscular spaces, which was indicative of poor tissue integrity.

### 3.4 High-quality peaks identified using ATAC-Seq and H3K27ac CUT&Tag

**Figure 3:**
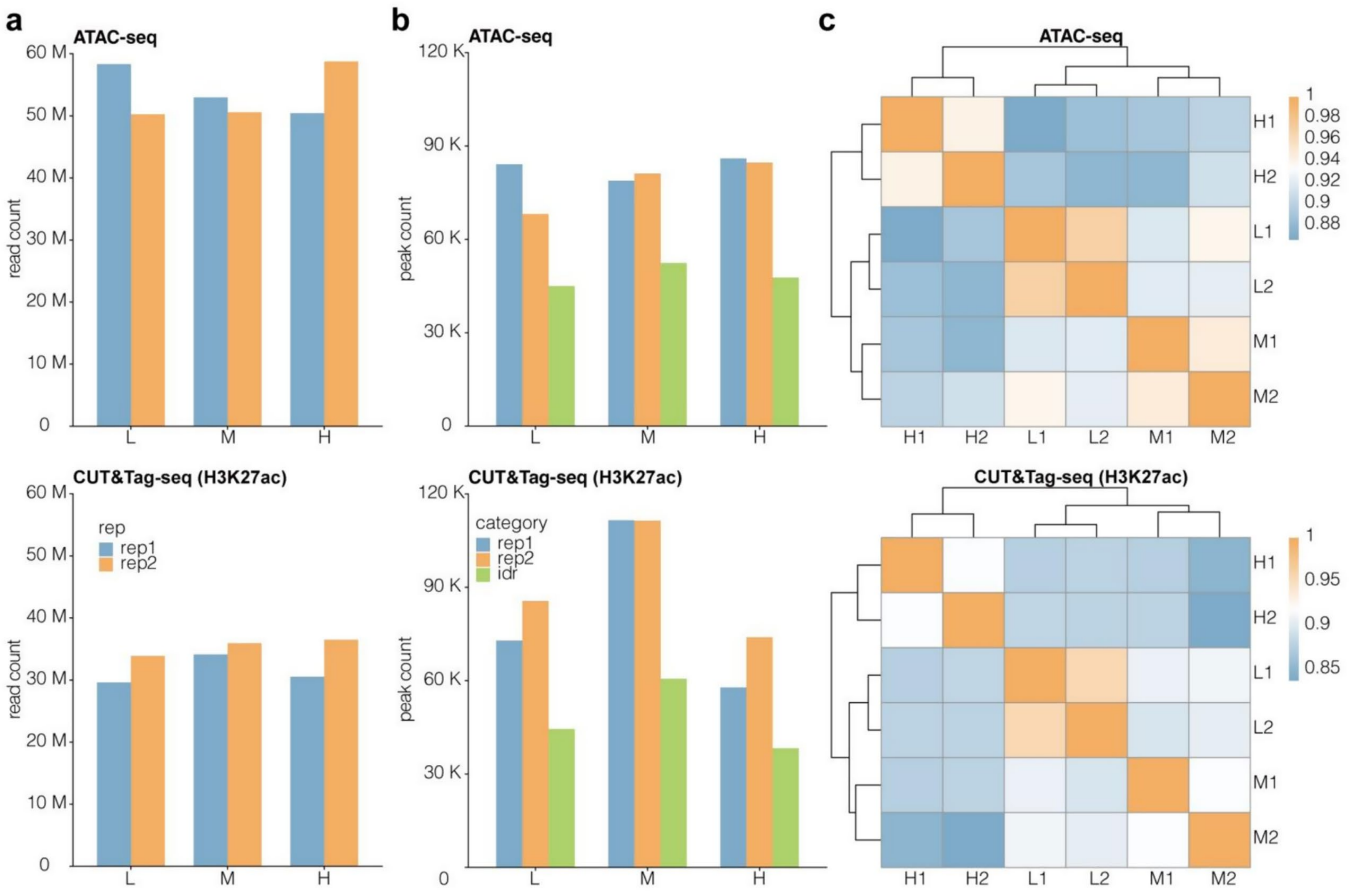
Quality assessment of epigenomics high-throughput sequencing data from Tibetan sheep muslce. Read count **(a)** and peak count **(b)** of CUT&Tag and ATAC-Seq data. M means one million, K means one thousand. **(c)** Similarity cluster for IDR peak signal with Pearson correlation coefficient (PCC) algorithm. Peak signal was normalized to transcripts per million (TPM).

We constructed the ATAC-Seq and CUT&Tag libraries of muscle tissues from Tibetan sheep fed diets containing three different NFC to NDF ratios (low, medium, and high). Raw fastq files were obtained after the high-throughput sequencing of these libraries. Multiple processing steps were performed, including read alignment, low-quality and duplicate read removal, peak calling, and peak read count calculation and normalization. The H3K27ac CUT&Tag data contained an average of 30 M reads/sample, while the ATAC-Seq data contained an average of 50 M reads/sample (Figure 3a). The sequencing depths of these samples were deemed to be adequate per ENCODE recommendations. About 60,000 to 110,000 CUT&Tag peaks and 70,000 to 80,000 ATAC peaks were identified (Figure 3b). Then, the irreproducible discovery rate (IDR) method was employed to screen for peaks with superb within-group consistency to ensure the reliability of the retained peaks. Finally, about 30,000 to 60,000 CUT&Tag IDR peaks and 40,000 to 50,000 ATAC-Seq IDR peaks were identified (Figure 3b). Subsequent analyses were based on these IDR peaks. Similarity analysis revealed that the data obtained via both methods showed excellent within-group repeatability and between-group heterogeneity (Figure 3c). These findings validated the high-quality nature of the high-throughput data obtained from the epigenomic analysis of Tibetan sheep muscle. The Pearson correlation coefficient (R ^2^) between biological replicates is shown in Table S1.

### 3.5 Heterogeneous genome-wide distribution of ATAC-Seq and H3K27ac CUT&Tag peaks

**Figure 4:**
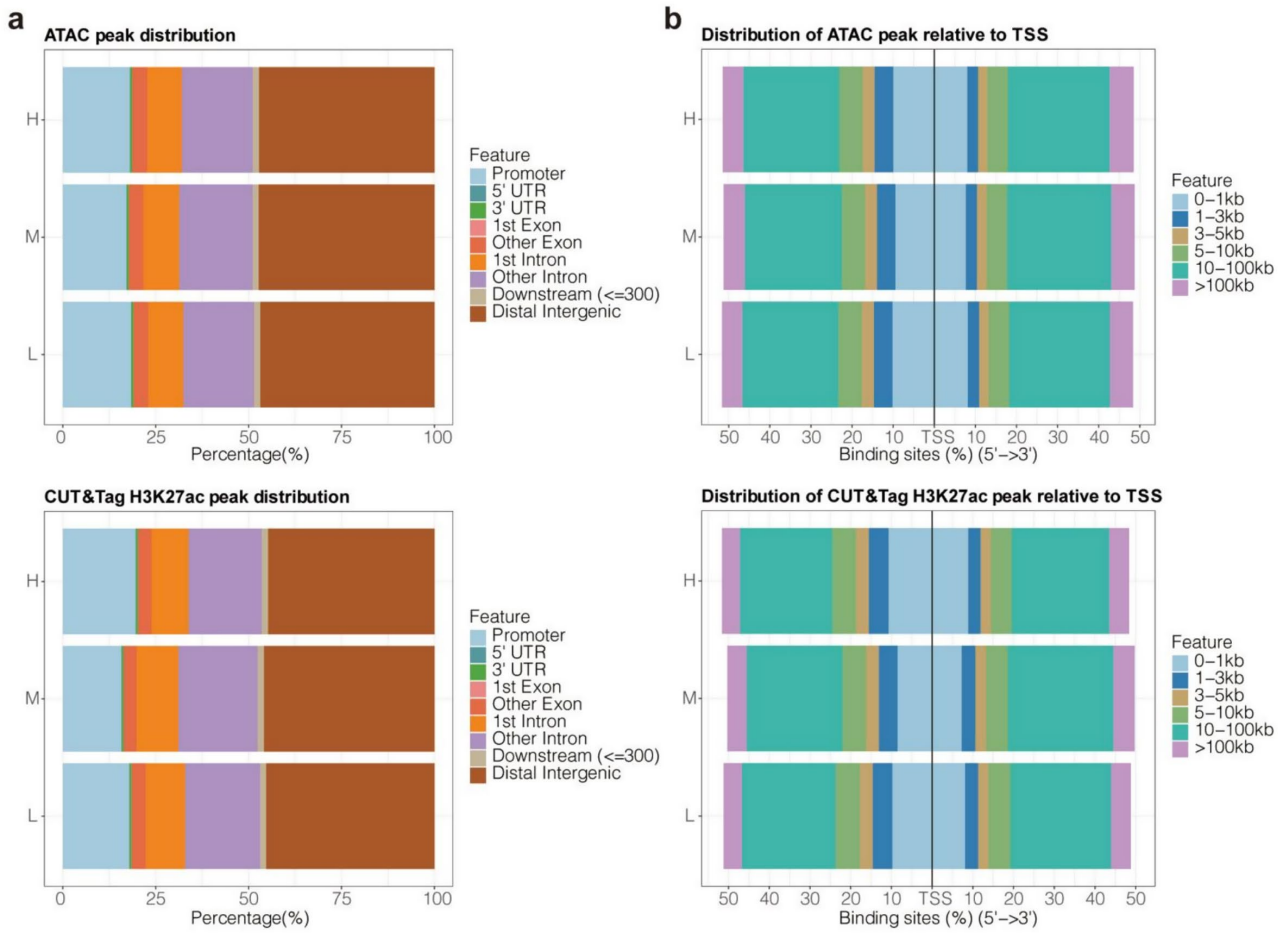
Genome-wide distribution bias of ATAC-Seq and H3K27ac CUT&Tag peaks. **(a)** The distributed proportion of peaks in 9 types of geometric features. **(b)** The distributed proportion of peaks in 6 types of distance ranges relative to TSSs.

The genome-wide distribution preferences of ATAC-Seq and H3K27ac CUT&Tag peaks were similar. Most of the peaks were distributed across non-coding regions, including promoters, 5′ untranslated regions (UTRs), 3’ UTRs, introns, and distal intergenic regions (Figure 4a). In particular, distal intergenic regions contained the majority of the ATAC and CUT&Tag peaks (50%), followed by promoters and introns other than the first intron (Figure 4a). This was consistent with previous evidence showing that enhancers are distal regulatory elements and are more abundant than promoters (each promoter generally corresponds to multiple enhancers). Specifically, about 50% of ATAC and CUT&Tag peaks were distributed in the distal regions (10–100 kb distance) of TSSs (Figure 4b). Meanwhile, only about 20% of the peaks were located in the proximal region (1–10 kb distance) of TSSs (Figure 4b). These results underscored the importance of distal CREs in epigenetic regulation in Tibetan sheep.

### 3.6 Sensitivity of enhances to changes in the NFC to NDF ratio

**Figure 5:**
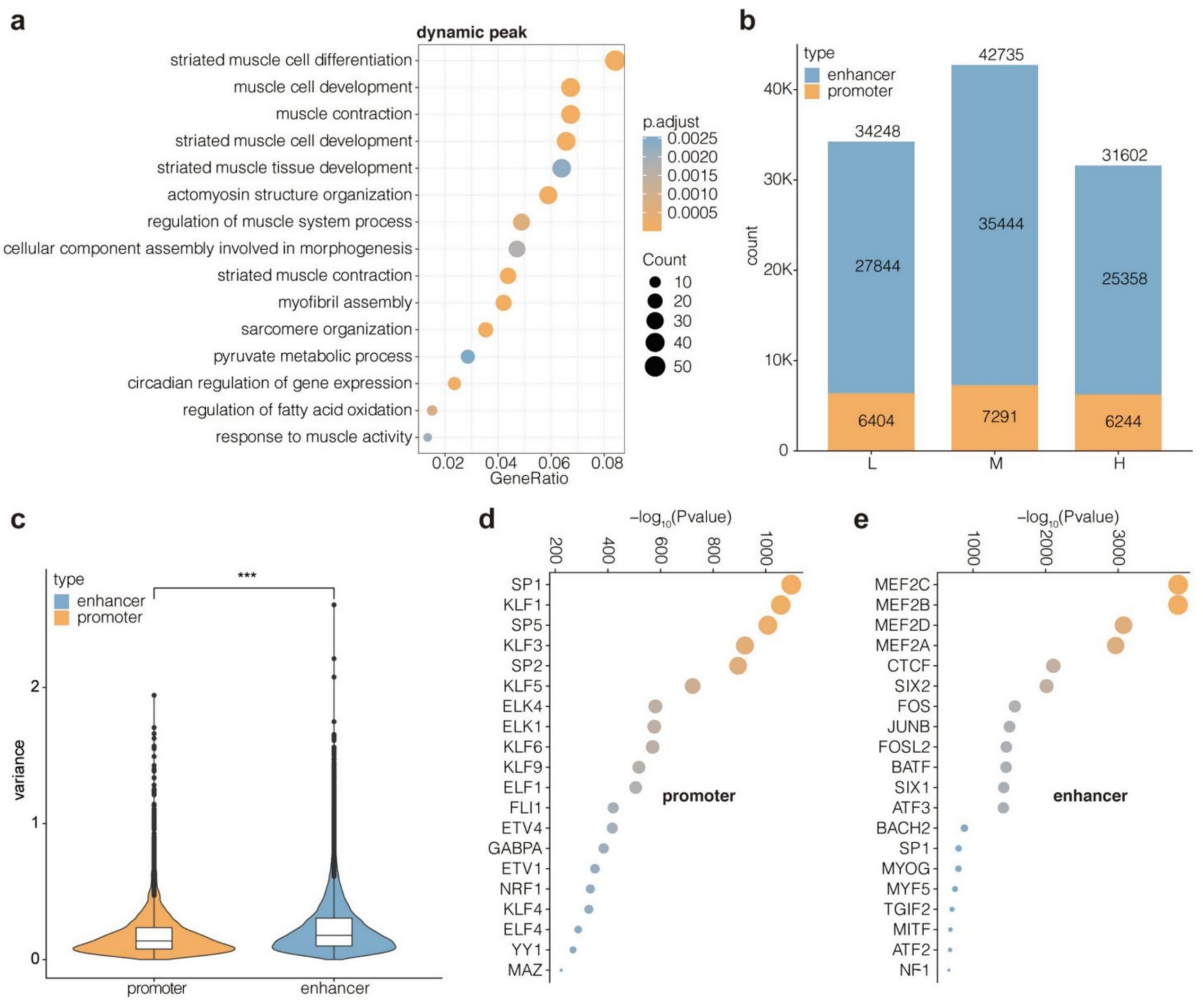
Identification and feature comparison of enhancers and promoters. **(a)** Enrichment for GO BP terms of dynamic peaks. Enhancers with the top 5,000 variance values are considered as dynamic peaks across group L, M, H. **(b)** Counts of promoters and enhancers in group L, M, H. **(c)** Signal variance values for promoters and enhancers across group L, M, H. Enrichment for TF motifs of promoters **(d)** and enhancers **(e)**. Dynamic peaks were defined as the top 5,000 enhancers showing highest variance (coefficient of variation >0.5) across groups L, M, and H. Promoters were classified as H3K27ac/ATAC-seq overlapping peaks within TSS ± 1.5 kb; enhancers were peaks beyond this range.

Since active promoters and enhancers are typically located in open chromatin regions, the H3K27ac CUT&Tag peaks overlapping with ATAC-Seq peaks were filtered for subsequent analysis to ensure the validity of H3K27ac peaks. Dynamic H3K27ac peaks were enriched in GO terms related to muscle development and fatty acid metabolism (Figure 5a), suggesting that changes in the NFC to NDF ratio affect these biological processes. Subsequently, H3K27ac peaks located near TSSs (1 kb upstream and 500 bp downstream) were classified as promoters, and the rest were classified as enhancers. The number of promoters (6404–7,291) in groups L, M, and H was relatively stable, while the number of enhancers (25,358–35,444) fluctuated greatly (Figure 5b). Additionally, the variance of enhancer signal intensity was significantly higher than that of promoter signal intensity among the three groups (*p* < 2.22e-16) (Figure 5c). Overall, the enhancers appeared to be more volatile than promoters, both in terms of number and signal strength. Combined with the GO annotation results, these findings indicated that the enhancers were more sensitive to changes in the NFC to NDF ratio than promoters. Indeed, the enhancers were enriched with multiple members of the *MRF* and *MEF2* family, including MEF2C, MEF2B, MEF2D, MEF2A, and MYOG. In contrast, promoters did not show such enrichment (Figures 5d,e).

### 3.7 Role of upregulated enhancers in muscle development

**Figure 6:**
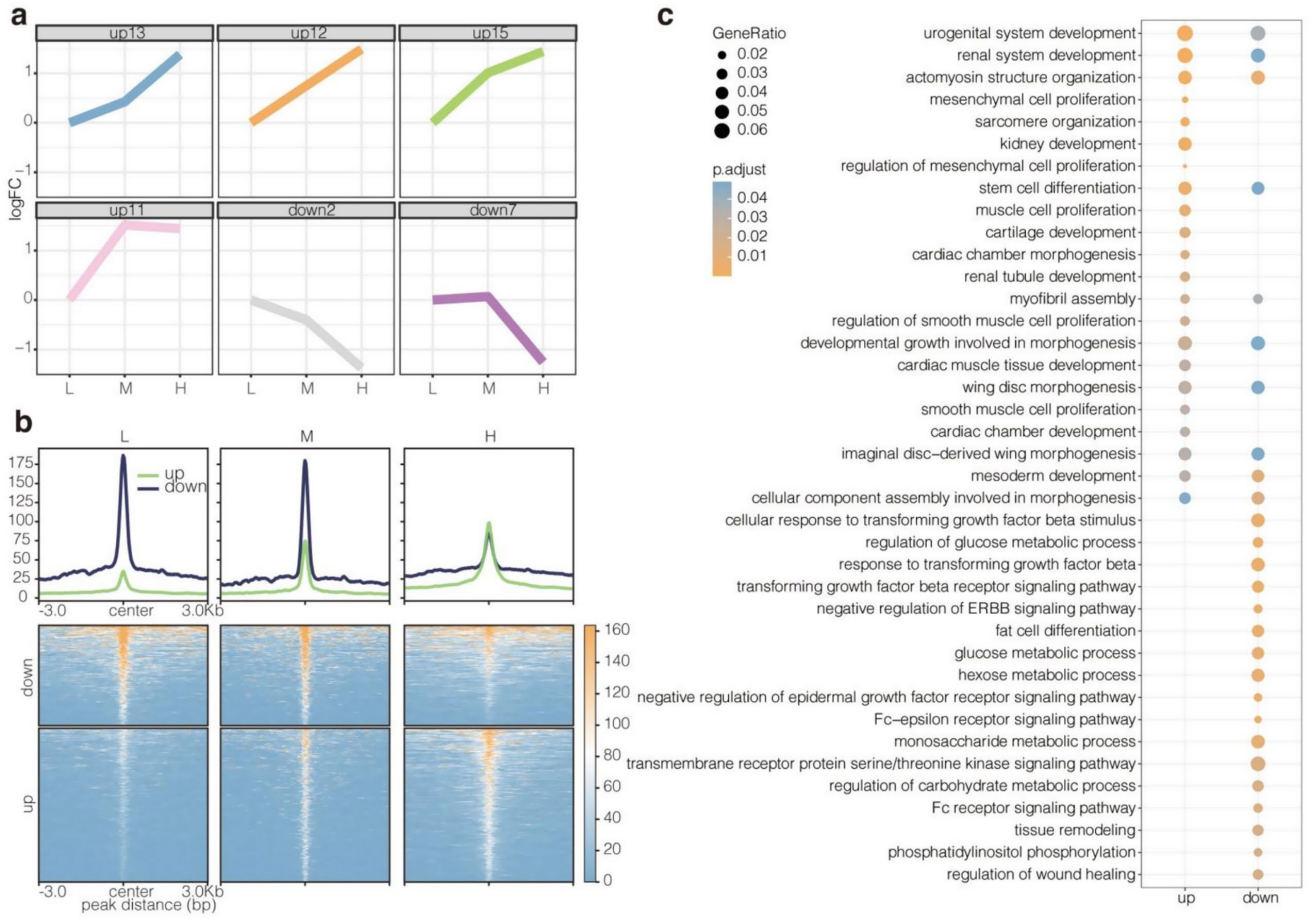
Identification and functional analysis of up- and downregulated enhancers. **(a)** Up- and downregulated enhancers were identified using stem software. STEM analysis used Group L (NFC/NDF = 0.71) as baseline control. Upregulated enhancers showed progressive signal increases (L → M → H), while downregulated enhancers exhibited signal decreases along this dietary gradient. **(b)** H3K27ac read intensity of up- and downregulated enhancers. **(c)** Enrichment for GO BP terms of up- and downregulated enhancers.

The enhancer signals in the three groups of samples were explored using STEM software, with Group L serving as the control/reference baseline representing the lowest NFC/NDF ratio (0.71). This provided a directional reference point for the analysis, where log2FC represents the log2(fold change) of enhancer signal levels relative to this baseline. Subsequently, four upregulated and two downregulated profiles were identified (Figures 6a,b). The upregulated profiles (curves 0–3) showed a progressive increase in H3K27ac signal levels as the NFC/NDF ratio increased across groups (L → M → H), indicating enhancer activation correlating with higher NFC/NDF ratios. Conversely, the downregulated profiles (curves 4–5) displayed the opposite pattern, with decreasing H3K27ac signal levels as the NFC/NDF ratio increased. The upregulated enhancers were highly enriched in the GO BP related to cell proliferation, differentiation, and organ development in muscles, including actomyosin structure organization, stem cell differentiation, muscle cell proliferation, and myofibril assembly (Figure 6c). Meanwhile, the downregulated enhancers were highly enriched in GO BP terms associated with basal metabolism, including glucose metabolic process, hexose metabolic process, and monosaccharide metabolic process (Figure 6c). These results suggested that the enhancers upregulated due to an increase in NFC to NDF ratios are involved in the regulation of muscle development. However, the downregulated enhancers do not show such involvement. Hence, the analysis of upregulated enhancers may reveal the effect of the NFC to NDF ratio on muscle performance in Tibetan sheep.

### 3.8 Enrichment of upregulated enhancers for MEF2 and SIX motifs

**Figure 7:**
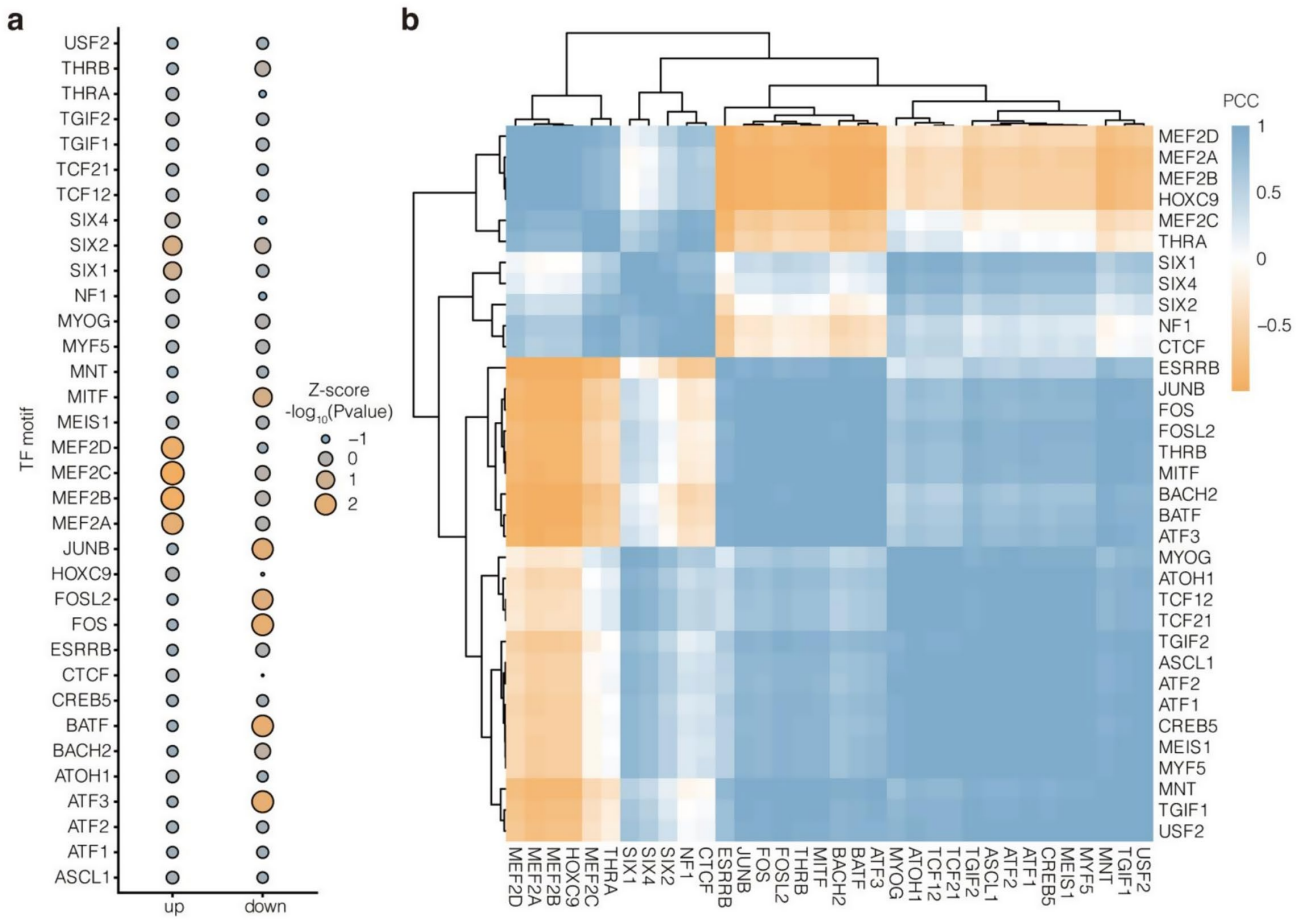
TF motif enrichment analysis of up- and downregulated enhancers. **(a)** Top 30 enriched TF motifs with the highest variance across up- and downregulated enhancers. -log10(*p* value) were scaled with Z-score algorithm. **(b)** Similarity cluster of these 30 TF motifs based on -log10(*p* value) with PCC algorithm.

Motif enrichment analysis showed that the up- and downregulated enhancers had different preferences for transcription factors (Figure 7a). Specifically, the upregulated enhancers were enriched for *MEF2* family members (*MEF2A, MEF2B, MEF2C,* and *MEF2D*) and *SIX* family members (*SIX1, SIX2,* and *SIX4*), which are closely involved in the regulation of muscle development. In contrast, the downregulated enhancers were specifically enriched for *MITF, JUNB, FOSL2, FOS, BATF*, and *ATF3*. These results suggested that upregulated enhancers may affect muscle performance in Tibetan sheep by recruiting *MEF2*s and *SIX*s in response to changes in the NFC to NDF ratio. In addition, transcription factor motifs with similar enrichment propensities showed clustering, which reflected their synergy. Among them, *HOXC9* and *THRA* were clustered together with *MEF2*s (*MEF2D*, *MEF2A, MEF2B*, and *MEF2C*). Meanwhile, *NF1* and *CTCF* were clustered together with *SIX*s (SIX1, *SIX4*, and *SIX2*). The results further demonstrated the potential regulators that mediate the effect of altered NFC to NDF ratios on muscle performance in Tibetan sheep (Figure 7b).

### 3.9 Transcriptomic analysis

The functional impact of epigenetic regulation on key muscle development genes was validated through RNA-seq transcriptome analysis, utilizing chromatin accessible regions identified by ATAC-Seq and transcription factor binding sites detected by CUT&Tag. During transcriptomic analysis, The range of raw reads for all samples is between 42.77 and 53.86 million, with an average sequencing depth of 47.78 million reads, providing sufficient data support for subsequent analysis. After quality control and filtering, the retention rate of effective readings (Clean_deads) for all samples is above 99%, indicating that the quality of the original data is high. The percentage of Q20 ranges from 97.41 to 98.41%, with an average of 97.99%, while the percentage of Q30 ranges from 92.7 to 95.18%, with an average of 94.12%. This indicates that the sequencing results have high accuracy, and the sequencing quality of the vast majority of bases is good. The GC content of the sample is relatively stable, ranging from 53.04 to 55.7%, with an average of 54.24%, which is close to the normal genomic GC content without significant deviation (Table 3).

**Table 3:** Comparison of quality indicators for sequencing different samples.

Items	Raw_reads	Clean_reads	Q20 (%)	Q30 (%)	GC (%)
L1	47.000098	46.678932	98.41	95.09	53.55
L2	51.005824	50.628714	98.38	95.11	55.09
L3	45.007044	44.656494	98.41	95.18	55.7
M1	43.924580	43.595780	97.73	93.49	54.1
M2	53.308038	52.883246	97.63	93.26	55.07
M3	53.858538	53.441162	97.41	92.7	53.04
H1	50.221886	49.815570	98.36	95.01	54.37
H2	42.773990	42.465834	97.9	93.88	53.47
H3	42.907606	42.597460	97.7	93.4	53.78

Raw_deads represents the raw sequencing reading (in millions), Clean_deads represents the filtered high-quality reading (in millions), Q20 (%) and Q30 (%) represent the percentage of bases with mass fractions ≥ 20 and ≥ 30, respectively, and GC (%) represents the percentage of G-C base content. L, M, and H represent different treatment groups, each containing three biological replicates.

**Figure 8:**
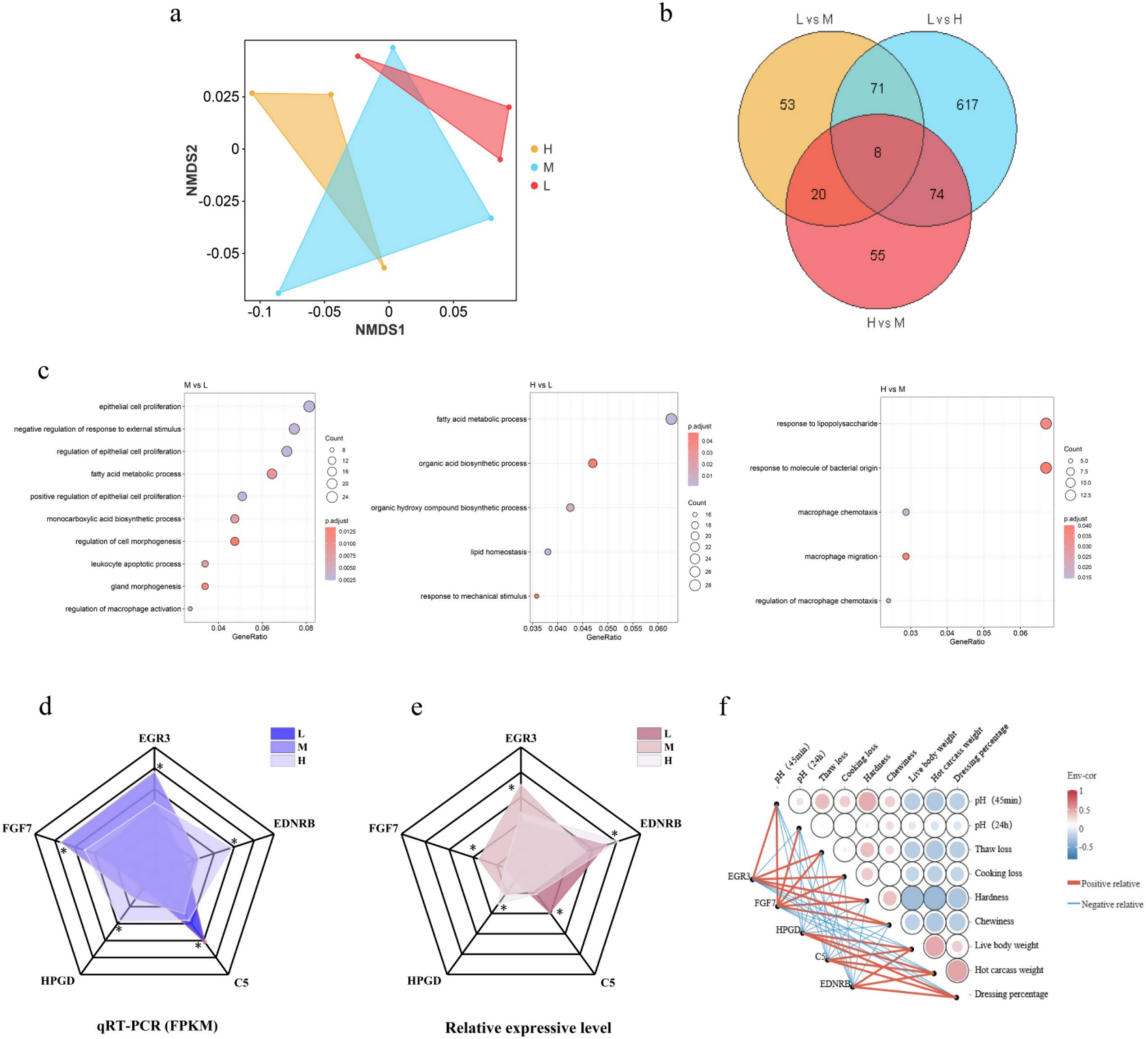
Transcriptomic analysis reveals differentially expressed genes associated with muscle development under varying dietary fiber compositions. **(a)** Non-metric multidimensional scaling (NMDS) analysis of transcriptome expression profiles. **(b)** Venn diagram analysis of commonly enriched genes across treatment comparisons. The diagram illustrates the overlap and unique distribution of differentially expressed genes among the three pairwise comparisons (H vs. M, H vs. L, and M vs. L) after filtering genes with missing values to ensure data integrity and comparability. **(c)** Gene Ontology (GO) enrichment analysis of differentially expressed genes. **(d)** Expression of DEGs from the sequencing results, * indicates *p* values < 0.05. **(e)** Expression of DEGs measured by RT-qPCR, * indicates *p* values < 0.05. **(f)** Correlation analysis. Pearson correlation coefficient was calculated to determine the relationship between core DEGs and phenotypic traits, including live weight, hot carcass weight, slaughter rate, pH measurement (45 min and 24 h after slaughter), thawing loss, cooking loss, hardness and chewiness.

NMDS analysis revealed distinct separation among experimental groups (Figure 8a), indicating significant differences in transcriptome expression patterns associated with varying ratios of non-fiber carbohydrates (NFC) and neutral detergent fiber (NDF). To ensure data comparability and validity, genes with missing values were excluded, and Venn diagrams were employed to illustrate commonly enriched genes across comparison groups (Figure 8b). Subsequently, GO enrichment analysis was conducted using these differentially expressed genes (Figure 8c), revealing significant enrichment in epithelial cell proliferation, fatty acid metabolic processes, and macrophage chemotaxis pathways, all of which are associated with muscle development. Integration of Venn diagram and GO enrichment analyses identified five core differentially expressed genes: *EGR3, FGF7, HPGD, C5,* and *EDNRB.*

To validate data reliability, RT-qPCR quantitative analysis was performed on these six genes (Figure 8d). The expression patterns of these genes demonstrated consistent up- or down-regulation trends, thereby confirming the reliability of RNA-Seq data. Correlation analysis revealed significant positive correlations between differentially expressed genes *HPGD*, *C5*, and *EDNRB* and live body weight, hot carcass weight, and dressing percentage. Differentially expressed genes *EGR3* and *FGF7* exhibited significant positive correlations with pH _45min_, pH_24h_, thaw loss, cooking loss, hardness, and chewiness (Figure 8e). The complete statistical data of each phenotype trait and differentially expressed gene pair, as well as the correlation statistics between each phenotype trait, are shown in Supplementary Tables S2, S3.

## 4 Discussion

Animal growth and development encompasses a complex integrated system characterized by structural and qualitative alterations in body tissues (15). This system effectively demonstrates the differential accumulation patterns of nutrients across various tissues and organs (16). It also serves as a crucial reference parameter for assessing feeding management and nutritional status in Tibetan sheep. These measurement parameters enable clear assessment of individual animal growth and development processes while facilitating enhanced production efficiency. In the present study, live weight, carcass weight, and dressing percentage were significantly enhanced when the NFC/NDF ratio reached 1.82. This suggests that elevated dietary NFC levels, characterized by increased rapidly fermentable carbohydrates, contribute to enhanced live weight in Tibetan sheep, consequently improving dressing percentage and carcass weight. These findings align with previous reports demonstrating that increased dietary concentrate levels enhance nutrient absorption and improve growth performance and feed conversion efficiency (17).

Meat physicochemical quality is closely correlated with dietary nutritional levels, with varying energy concentrations effectively enhancing meat quality (18). pH variations are highly correlated with muscle glycogen reserves (19). In the present experiment, pH_45min_ values decreased significantly with increasing NFC/NDF ratios, whereas no significant differences were observed among the three groups at pH_24h_. Previous studies have demonstrated that lactic acid accumulation during glycogen breakdown reduces muscle pH, with substrate availability and glycolytic enzyme activity synergistically regulating the rate and extent of this process (20). This suggests that elevated NFC/NDF ratios may promote fast-glycolytic muscle fiber development, thereby accelerating Tibetan sheep growth, as corroborated by live weight data. Thaw loss and cooking loss serve as indicators of muscle water-holding capacity and muscle fiber structural integrity (21). Muscle fiber development determines cell membrane system integrity, with mature fibers exhibiting well-developed sarcoplasmic reticulum and mitochondrial structures that maintain optimal intracellular and extracellular water balance (22). At an NFC/NDF ratio of 1.82, water loss during thawing and cooking was significantly reduced, indicating enhanced muscle fiber development with optimal myofibrillar spacing that creates ideal water retention capacity. Hardness and chewiness are fundamentally determined by muscle fiber type differentiation and maturation during muscle development (23). With increasing NFC/NDF ratios, muscle hardness and chewiness exhibited significant decreasing trends, indicating effective enhancement of meat tenderness. These results align with the muscle developmental characteristics revealed in previous experiments, further confirming that nutritional regulation strategies modulate meat quality characteristics through alterations in muscle fiber development patterns.

Histological analysis provides a comprehensive morphological assessment of muscle tissues in ruminants and is a crucial tool for evaluating skeletal muscle growth and development (24). In this study, optimal skeletal muscle growth was detected when the dietary ratio of NFC to NDF was 1.82, as indicated by tightly organized fibers, minimal intermuscular gaps, and a increased cross-sectional area of muscle fibers. This suggests that a proper balance of dietary energy sources is crucial for skeletal muscle growth and development. One potential explanation is that NFC (e.g., starch) serve as the primary energy source for rumen microbial fermentation, while NDF (e.g., cellulose) plays a key role in promoting the structural development of the rumen (25). When the ratio of NFC to NDF is balanced, there are sufficient substrates for rumen microbial growth and adequate rumen development. Additionally, nutrients such as volatile fatty acids produced via rumen fermentation can be absorbed by the host, providing energy and raw materials for the growth of skeletal muscle and other tissues (26). The findings of the present study support this hypothesis. Notably, we also observed that as the dietary NFC to NDF ratio decreased, muscle histological parameters declined, leading to reduced fiber density, smaller fiber diameter, and increased intermuscular gaps. This suggests that low amounts of dietary NFC may restrict anabolic activity in skeletal muscle tissue, impairing normal growth. This highlights the importance of balancing dietary energy sources. The growth and development of skeletal muscle in ruminants is a complex biological process that is influenced not only by nutritional factors but also by genetic backgrounds, endocrine regulation, and physical activity (27). Therefore, elucidating the intrinsic relationship between dietary energy sources and skeletal muscle growth requires an integration of research in the fields of molecular biology, biochemistry, and other disciplines and subsequent validation in larger ruminant populations.

To further investigate the regulatory effects of nutritional factors on skeletal muscle growth and development, we systematically analyzed the epigenetic landscape of skeletal muscle in Tibetan sheep. We adopted a novel integrated approach that combines ATAC-seq and H3K27ac CUT&Tag to decode the complex mechanisms of diet-induced muscle development (28). Our findings revealed a comprehensive epigenetic trajectory involving the regulation of muscle progenitor cells, generating an innovative framework to identify the key regulatory elements that drive muscle-specific gene expression patterns under varying NFC/NDF ratios (29). To our knowledge, this study is the first to perform a comprehensive epigenomic analysis of diet-induced muscle development in Tibetan sheep, offering valuable insights into the fundamental mechanisms of muscle plasticity induced by nutritional interventions and providing a valuable resource for livestock muscle biology research.

This study generated high-quality sequencing data, with sequencing depths of 50 M for the ATAC-seq library and 30 M for the CUT&Tag library, greatly surpassing ENCODE standards and providing adequate sequencing depth. Through peak calling, 60,000–110,000 CUT&Tag peaks and 70,000–80,000 ATAC peaks were identified, indicating the presence of numerous potential cis-regulatory elements in the Tibetan sheep muscle genome, consistent with the regulatory characteristics of mammalian genomes (30). The large number of peaks provides a wealth of candidate sites for exploring the regulatory mechanisms of muscle development in Tibetan sheep. Finally, the IDR method was employed to filter peaks with high intra-group consistency, effectively eliminating noise signals and batch effects. As a result, 30,000–60,000 high-confidence CUT&Tag peaks and 40,000–50,000 high-confidence ATAC peaks were obtained, providing a solid database for subsequent analyses.

Notably, the genome-wide distribution patterns of ATAC-Seq and H3K27ac CUT&Tag peaks were found to be similar, with both sets of peaks primarily located in the non-coding regions of the genome. Previous studies have shown that 54% of human ATAC-seq peaks are located in regions distal to promoters, highlighting the ubiquity and importance of distal regulation in gene regulation (31). These non-coding regions are rich in transcription factor binding sites and histone modifications, which play a crucial role in cell-specific gene expression (32). In the present study, consistent results were observed in the Tibetan sheep muscle genome, suggesting that distal cis-regulatory elements play a critical role in regulating skeletal muscle development in these animals. Interestingly, approximately 50% of the ATAC and CUT&Tag peaks were concentrated in the distal regions 10–100 kb upstream and downstream of the TSS, while only about 20% were located in the proximal regions (1–10 kb from the TSS). This preferential distribution underscores the significance of distal regulatory elements, further supporting the aforementioned hypothesis. Current evidence indicates that the median distance between enhancers and their target genes is 10–100 kb (33), which closely aligns with the results of the present study. Enhancers, particularly those in distal intergenic regions, are known to play a crucial role in fine-tuning gene expression, often by interacting with multiple promoters to coordinate the activation of tissue- and condition-specific genes (34). Each promoter in the mammalian genome is typically associated with more than 10 enhancers, collectively forming a finely tuned regulatory network (35). This study is the first to confirm the widespread presence of enhancers in the genome of Tibetan sheep. These findings support the hypothesis that distal regulatory elements play key roles in the epigenetic regulation of meat quality-related traits in Tibetan sheep, potentially influencing their adaptation to different environmental conditions.

In this study, GO enrichment analysis revealed that upregulated enhancers were predominantly enriched in biological processes related to muscle development. This suggests that muscle development-related enhancers become increasingly active as the dietary NFC/NDF ratio rises. It is important to note that these GO pathways are interconnected. Previous studies have demonstrated that in the actomyosin structure organization pathway, actin a crucial cytoskeletal component is spatiotemporally regulated through the tissue-specific expression and subcellular localization of Rho GTPase regulators (36). Nowak et al. elucidated the role of actin in muscle morphogenesis and myofibril assembly, demonstrating that actin not only supports cell morphology and structure but also regulates cell movement and function through dynamic polymerization and depolymerization. Therefore, it plays a role in promoting muscle tissue development and maturation (37). These findings are consistent with the results of the present study, which showed that the activity of actin-related enhancers significantly increases as the dietary NFC/NDF rises, likely promoting myofibril assembly and muscle development. Notably, Du et al. demonstrated that nutrition can influence fetal skeletal muscle development, with nutritional factors affecting muscle development by regulating actin expression and activity (38). This could explain the relationship between the dietary NFC/NDF ratio and the activity of muscle development-related enhancers at the molecular level.

Interestingly, our motif enrichment analysis revealed that upregulated enhancers were specifically enriched with binding sites for the *MEF2* and *SIX* family transcription factors associated with muscle development. This suggests that in muscle tissue, the expression of downstream genes may be regulated via the dynamic recruitment of these key transcription factors at enhancer regions in response to nutritional interventions, thereby influencing muscle traits in sheep. This hypothesis is in line with the findings reported by Zhang et al., who found that super-enhancers (SEs) with sustained high expression in pig muscle are enriched with binding sites for key muscle-regulating factors like *MYOD* and *MYOG*. This suggests that SEs may sustain the expression of skeletal muscle genes by recruiting these factors (39). Notably, as the NFC/NDF ratio in the diet increased in our study, the enrichment of *MEF2* and *SIX* family transcription factor binding sites in sheep muscle tissue also gradually increased. *MEF2* family members act as crucial regulators of muscle differentiation, activating muscle-specific genes and promoting the terminal differentiation of myogenic cells through cooperation with muscle-regulating factors such as MyoD (40). This aligns with the findings reported by Lyu et al., who analyzed histone H3K27ac modifications during bovine satellite cell differentiation. Their results revealed that the active enhancer regions of satellite cells during differentiation were enriched with binding sites for *MEF2*, *MyoD*, *AP-1*, and other transcription factors, which collectively coordinated the regulation of muscle gene expression (10). Interestingly, studies have shown that *SIX* family transcription factors play a crucial role in regulating muscle satellite cell proliferation and differentiation (41). Wang et al. demonstrated that *SIX4* regulates skeletal muscle development, and its *SIX* domain and *SIX*-type homeodomain are essential for binding to specific DNA sequences and protein–protein interactions. Notably, the *SIX4* protein is involved in activating the expression of myosin genes during somite development and regulates myogenesis by binding to the *MEF3* site on the myosin promoter (42). Increasing the dietary NFC/NDF ratio allows Tibetan sheep to obtain higher amounts of energy, promoting the synthesis of short-chain fatty acids in the rumen (43). Studies by Sean and Mark also demonstrated that elevated energy levels enhanced MEF2 transcriptional activity and its DNA binding capacity (44). Therefore, we hypothesize that elevated dietary NFC/NDF ratios provide increased readily available energy, alter metabolic status, and activate key transcription factors including *MEF2* and *SIX*, thereby regulating skeletal muscle growth and development in Tibetan sheep.

A clustering of transcription factor motifs with similar enrichment patterns was observed in this study. For instance, *NF1* and *CTCF* were closely clustered with the SIX family transcription factors (*SIX1*, *SIX2*, and *SIX4*), while *HOXC9* and *THRA* were clustered with the *MEF2* family transcription factors (*MEF2A, MEF2B, MEF2C,* and *MEF2D*). This clustering was particularly observed during muscle cell differentiation, where these transcription factors cooperate to regulate muscle-specific genes and maintain stable muscle phenotypes. Previous studies have demonstrated that *MEF2* transcription factors can cooperate with *HOXC9* and *THRA*, particularly during muscle cell differentiation, jointly regulating muscle-specific genes to maintain stable muscle phenotypes (45). At the molecular level, *HOXC9* and *THRA* function as co-transcriptional factors that form transcriptional complexes with *MEF2* through protein–protein interactions, synergistically activating structural protein genes including myosin heavy chain (MyHC), actin, and troponin, which directly determine muscle contraction velocity, force generation, and metabolic properties, consequently enhancing muscle pH and water-holding capacity (46–48). Meanwhile, the clustering of NF1 and CTCF with *SIX* transcription factors was indicative of a cross-factor synergistic regulatory interaction among these transcription factors. NF1 can promote the binding of *CTCF* and *SIX* transcription factors to specific enhancer regions by modulating signaling pathways and altering genome structure, thereby enhancing the expression of muscle development genes (45). Additionally, NF1 and *CTCF* may also alter the three-dimensional structure of DNA, which could enable them to cluster at specific enhancer regions, thereby enhancing the precision and responsiveness of gene expression (49). Hence, these transcription factors may jointly regulate the activity of enhancer regions. This is consistent with the findings reported by Whyte et al., who found that transcription factors cooperate to bind to SE regions while regulating cell identity genes (50), Therefore, we hypothesize that these enriched transcription factors may collectively regulate enhancer activity through potential co-regulatory relationships during Tibetan sheep muscle development, consequently influencing muscle-specific gene expression and enhancing growth performance and muscle physicochemical properties in Tibetan sheep.

To validate the regulatory effects of key enhancers *MEF2* and *SIX* family on muscle development, high-throughput RNA sequencing analysis was performed on Tibetan sheep muscle tissues to explore underlying mechanisms through key regulatory genes and signaling pathways. Myogenesis encompasses embryonic muscle development, myogenic progenitor cell proliferation, myoblast proliferation and differentiation, and muscle maturation (51, 52). The present study revealed that key pathways, including epithelial cell proliferation, fatty acid metabolic process, and macrophage chemotaxis, are associated with muscle development and meat quality. Previous studies have demonstrated that within the fatty acid metabolic process pathway, fatty acids and their metabolites influence muscle development through regulation of myotube formation and myofibrillar fiber types (53). Additionally, myocytes, as specialized epithelial cells, undergo proliferation and differentiation processes regulated by lipid metabolism (54). The key gene *HPGD*, enriched in this pathway, modulates meat quality characteristics through regulation of fatty acid metabolic interactions (55). Studies have demonstrated that *HPGD* influences calcium homeostasis, protein degradation, and membrane integrity through degradation of prostaglandin E2 and F2α, thereby regulating meat tenderness, water-holding capacity, and flavor (56). Additionally, *HPGD* interacts with the *PPAR*s signaling pathway to regulate intramuscular fat deposition, consequently affecting meat marbling (57). The present study confirmed through correlation analysis that *HPGD* exhibits strong correlation with meat quality traits.

The macrophage chemotaxis pathway initiates and coordinates cellular processes during muscle repair, with this regulatory mechanism determining the temporal sequence and quality of muscle regeneration (58). This pathway promotes clearance of necrotic tissue through pro-inflammatory M1 macrophages, subsequently transitioning to anti-inflammatory M2 macrophages that facilitate tissue reconstruction (59). *EDNRB* regulates macrophage polarization between M1 (pro-inflammatory) and M2 (anti-inflammatory) phenotypes (60). The endothelin-*EDNRB* axis modulates macrophage chemotactic capacity and regulates their recruitment to muscle injury sites. *EDNRB* expression was significantly upregulated when the dietary NFC/NDF ratio reached 1.82. Notably, the differentially expressed gene C5 within this pathway undergoes cleavage to generate C5a and C5b (61). C5a directs macrophage migration to inflammatory or injury sites through C5aR receptor binding, inducing pro-inflammatory cytokine production (62), while C5 expression was downregulated with increasing NFC/NDF ratios. Correlation analysis revealed positive correlations between C5, *EDNRB*, and Tibetan sheep growth performance, indicating that macrophage-mediated reduction of inflammatory responses and enhanced transition to anti-inflammatory states are crucial for muscle development and overall growth in Tibetan sheep.

Within the epithelial cell proliferation pathway, the promoter region of differentially expressed gene EGR3 contains multiple A/T-rich MEF2 binding sites (63). The differentially expressed gene *FGF7* contains *MEF2* recognition elements where *MEF2* co-binds with histone-modifying enzyme p300, facilitating chromatin accessibility within the *FGF7* expression locus (64). Therefore, we hypothesize that the differentially expressed genes *EGR3* and *FGF7* are downstream targets of the *MEF2* enhancer. Previous studies have demonstrated that the *MEF2-FGF7* signaling axis promotes satellite cell activation during muscle development and regeneration (65). Notably, *MEF2* establishes a classical negative feedback loop whereby *MEF2* activates *HDAC9* expression during early muscle differentiation, while *HDAC9* subsequently binds *MEF2* to suppress its transcriptional activity during late differentiation, thereby forming a self-regulatory circuit (66). In the present study, decreased expression of *EGR3* and *FGF7* at an NFC/NDF ratio of 1.82 was consistent with the negative feedback mechanism governing *MEF2* transcriptional regulation.

These findings demonstrate that with increasing dietary NFC/NDF ratios, the differentially expressed genes and enriched pathways are closely associated with muscle development, further confirming the pivotal role of *MEF2* and *SIX* family transcription factors in nutrition-mediated muscle development. However, the transcriptomic analysis in the present study has inherent limitations. First, RNA-seq reflects gene expression profiles at specific time points and cannot fully capture the dynamic temporal changes in gene expression. Second, although correlations between enhancer activity and gene expression were observed, establishing definitive causal relationships remains challenging and requires additional functional validation experiments.

Despite these limitations, our findings provide actionable insights for practical application in Tibetan sheep production. Based on our experimental results, we recommend an NFC/NDF ratio of 1.82 for 2–5 month-old growing lambs to optimize muscle development and meat quality, as evidenced by the significant improvements in live weight, carcass weight, and meat tenderness observed in our study. This dietary ratio should be implemented for at least 60 days to achieve measurable improvements in muscle fiber architecture and meat physicochemical properties. The diet composition achieving this optimal ratio (40.5% corn, 14.13% rapeseed meal, 6.39% cottonseed meal with 15% each of oat hay and silage, as detailed in Table 1) can serve as a reference formulation for commercial feed mills. These recommendations provide a practical framework for implementing precision nutrition strategies in intensive Tibetan sheep production systems, bridging the gap between molecular mechanisms and field application.

To further validate and expand upon these findings, future studies should employ CRISPR-dCas9 to validate MEF2/SIX enhancer functions, Hi-C to map enhancer-promoter interactions, and metabolomics to link epigenetic changes with muscle metabolic remodeling. Such investigations would not only confirm the causal relationships suggested by our correlative data but also refine the dietary recommendations for different production contexts and sheep breeds.

## 5 Conclusion

**Figure 9:**
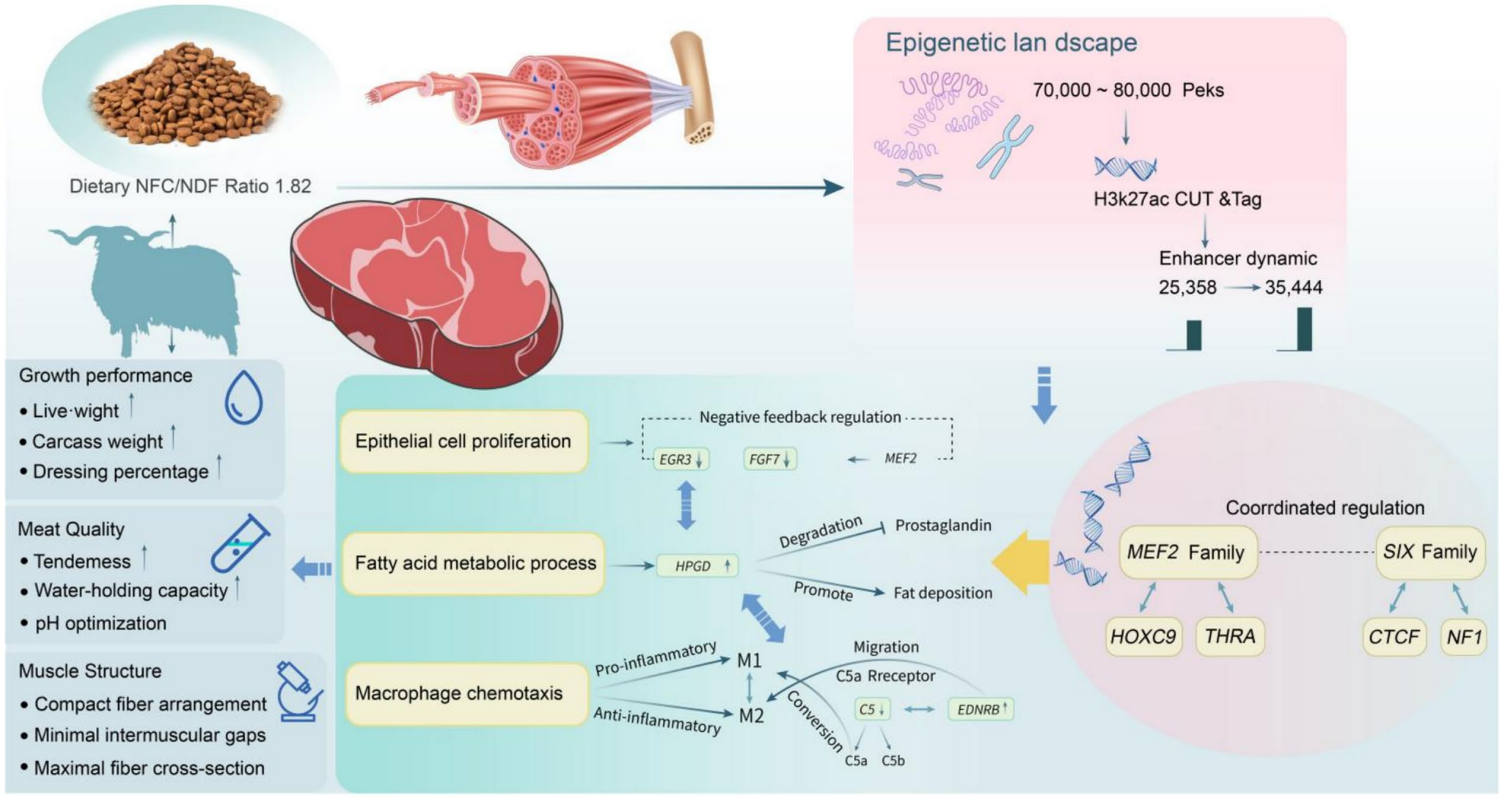
Proposed molecular mechanism of NFC/NDF ratio-mediated skeletal muscle development in Tibetan sheep through epigenetic regulation.

This study integrated multi-omics data to investigate the epigenetic heterogeneity in the skeletal muscle of Tibetan sheep under different dietary ratios of NFC to NDF. An NFC/NDF ratio of 1.82 yielded the most compact muscle fiber structure, minimal intermuscular spacing, and optimal growth performance. Epigenomic analyses highlighted the importance of distal regulatory elements, particularly enhancers, in this process. The enhancers upregulated in response to increasing NFC/NDF ratios appeared to influence skeletal muscle development in Tibetan sheep by recruiting *MEF2* and *SIX* family members. RNA-seq analysis further corroborated the impact of key enhancers on skeletal muscle development at the transcriptomic level. These findings reveal the molecular mechanisms mediating the effect of energy balance on skeletal muscle growth and development in Tibetan sheep, providing novel perspectives for enhancing feed efficiency and meat quality in precision livestock farming (Figure 9). Future studies should validate the regulatory effects of key enhancers on target genes and identify the synergistic effects of transcription factors.

## Data Availability

The raw sequence data reported in this paper have been deposited in the Genome Sequence Archive (Genomics, Proteomics & Bioinformatics 2021) in National Genomics Data Center (Nucleic Acids Res 2022), China National Center for Bioinformation/Beijing Institute of Genomics, Chinese Academy of Sciences (GSA: CRA023329) that are publicly accessible at https://ngdc.cncb.ac.cn/gsa.

## Supplementary material

The Supplementary material for this article can be found online at:

https://www.frontiersin.org/articles/10.3389/fnut.2025.1658319/full#supplementary-material
